# Hyaline vascular variant of unicentric Castleman disease of the tonsil: a case report

**DOI:** 10.1186/s13000-019-0836-y

**Published:** 2019-06-29

**Authors:** Ping Li, Huaipu Liu, Hao Li, Ang Li, Guangyin Yu, Weihua Yin

**Affiliations:** 1 Department of Pathology, Shenzhen Hospital of Peking University, 1120 Lianhua road, Shenzhen, 518036 China; 2 Department of Cardiothoracic Surgery, Shenzhen Children’s Hospital, 7019 Yitian road, Shenzhen, 518038 China

**Keywords:** Castleman disease (CD), Tonsil, Hyaline vascular variant, Unicentric Castleman disease (UCD), Lymphoproliferative disorder

## Abstract

**Background:**

Castleman disease (CD) is a lymphoproliferative disorder with an unknown etiology. The disease may be unicentric (UCD) or multicentric (MCD), and three histopathologic variants have been described: hyaline vascular (HV), plasma cell (PC), and mixed type. Extranodal CD is rare. Herein, we report a case of CD presenting as a tonsillar mass, which has not been documented in the literature.

**Case presentation:**

The patient was a 32-year-old man. Laryngoscopy revealed tonsillar hypertrophy, and the patient underwent a low-temperature plasma tonsillectomy. Microscopic examination of permanent sections showed lymphoid follicular hyperplasia, a portion of which appeared to be a fusion of nodular hyperplasia (composed of lymphoid follicles of variable size and shape). These distinctive follicles with atrophic hyalinized germinal centers and a broad mantle zone of small lymphocytes formed concentric rings (so-called onion-skin arrangement). Medium-sized vessels and a plethora of capillaries were present in the center of the lymphatic follicles, mantle zones, and interfollicular areas. A characteristic lollipop appearance was also observed due to the onion-skin arrangement of the expanded mantle zone lymphocytes with a vessel penetrating the germinal center. No aberrant lymphoid population was present based on CD3, CD5, CD20, CD79α, CD21, CD23, bcl-2, cyclin D1, and ki-67 immunostaining. Tests for human herpesvirus (HHV)-8 and Epstein Barr virus (EBV)-encoded small RNA (EBER) were negative. Therefore, a diagnosis of an HV variant UCD was rendered. The patient was treated by local excision without any other therapy based on the diagnosis. At the 7-month follow up, the patient had no recurrent symptoms or masses.

**Conclusion:**

We present an unusual case of a tonsil presenting hyaline vascular Castleman disease (HVCD). This study aims to highlight CD as a differential diagnosis that should be considered by otolaryngologists and pathologists for lymphoproliferative disorders of the tonsil.

## Background

Castleman disease (CD) is an uncommon benign lymphoid hyperplasia with several clinical and morphologic variants that is also known as giant lymph node hyperplasia, angiofollicular lymphoid hyperplasia, angiomatous lymphoid hamartoma, and follicular lymphoreticuloma.

In 1954, this disease was first described by Dr. Benjamin Castleman in a patient with few or no symptoms but with solitary mediastinal lymphadenopathy [1]. These features were so distinctive that the type of disease was later characterized as unicentric Castleman disease (UCD). In 1978, Gaba et al. described a patient with multiple retroperitoneal and axillary lesions that were histologically similar to those of Dr. Castleman’s patient, thus providing the first example of multicentric Castleman disease (MCD). Therefore, CD is categorized into two clinical variants: UCD and MCD [2]. UCD presents as a solitary mass that predominantly occurs in the mediastinal, retroperitoneal, and cervical lymph nodes, and it is not typically associated with generalized symptoms. In contrast, MCD often involves systemic symptoms (e.g., fever, pleural effusion, asthenia, ascites), including POEMS syndrome, as well as a poorer prognosis [2]. This syndrome is a rare paraneoplastic syndrome caused by clones of aberrant plasma cells (PCs) and affects many other parts of the body. Each letter in the word “POEMS” stands for the following signs and symptoms: polyneuropathy, organomegaly, endocrinopathy, monoclonal gammopathy, and skin changes [3]. Three pathological subtypes of CD have been identified: hyaline vascular (HV), PC, and mixed. Of the three histological variants, the hyaline vascular Castleman disease (HVCD) type accounts for 90% of cases and most commonly presents as a mediastinal nodal mass [4]. While HVCD predominantly occurs in younger individuals who show few systemic symptoms, plasma cell Castleman disease (PCCD) is observed in an older population, often with systemic involvement. A mixed subtype has been reported in a few patients [3]. The clinical manifestations and management of the disease are distinct for different clinical and pathologic subtypes of CD.

In recent years, interest in CD has increased, as it has been associated with a variety of malignancies, including Kaposi’s sarcoma (KS), non-Hodgkin’s lymphoma, Hodgkin’s lymphoma, and follicular dendritic cell sarcoma, as well as with human herpesvirus (HHV)-8 and human immunodeficiency virus (HIV) [5–7].

Although CD can occur wherever lymphoid tissue is present, it usually presents as a solitary mediastinal nodal mass [2]. Solid organ involvement is rare, and isolated tonsil involvement is extremely rare. Here, we report a case of HVCD presenting as a tonsillar mass, which has not been documented in the literature. We aim to suggest CD as a differential diagnosis for lymphoproliferative disorders in the tonsil that should be considered by otolaryngologists and pathologists.

## Case presentation

### Clinical history

A 32-year-old man with tonsillar hypertrophy detected during a physical examination was referred to us. The physical symptoms first appeared three years prior. No inciting events were associated with the appearance of tonsillar hypertrophy. His vital signs were as follows: body temperature 36.5 °C, pulse 78 beats per minute, respiratory rate of 18 breaths per minute, and blood pressure 120/79 mmHg. His physical examination revealed nonspecific findings with the exception of tonsillar hypertrophy. He had no signs or symptoms of an autoimmune disease. His family history did not suggest the presence of any familial disease. No lymphadenopathy, POEMS syndrome, lymphoma, or other cancers were present. Tests were negative for anti-HCV antibody, treponema pallidum-specific antibody (TP-Ab) and HIV antigen/antibody. The test results for HBV indicators were as follows: HBsAg 0.23 (negative), HBsAb 30.78 (positive), HBeAg 0.38 (negative), HBeAb 0.23 (negative), and HBcAb 1.85 (negative). Other laboratory tests also revealed no abnormal findings.

**Fig. 1:**
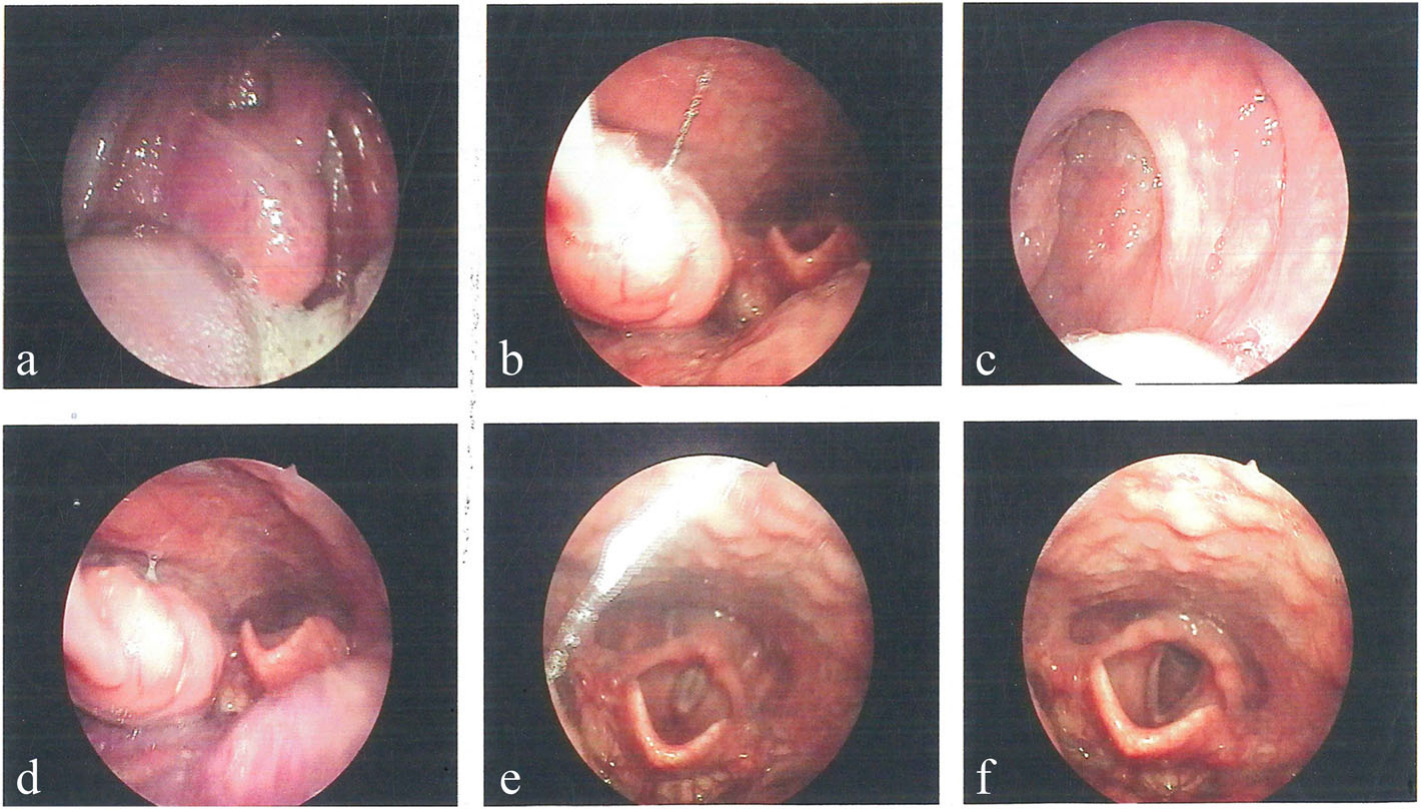
Laryngoscopy results. **a**-**f** Images of endoscopy findings showing tonsillar hypertrophy (right, grade 3; left, grade 2); an elongated uvula; and posterior pharyngeal wall lymphoid hyperplasia

Laryngoscopy revealed the following: tonsillar hypertrophy (right, grade 3; left, grade 2), an elongated uvula, and posterior pharyngeal wall lymphoid hyperplasia (Fig. 1a-f). The nasopharynx was smooth and symmetrical. Based on the physical examination and related laboratory tests, the initial diagnosis were tonsil hypertrophy and chronic tonsillitis. The patient underwent a low-temperature plasma tonsillectomy under general anesthesia. Two lesions were sent for pathological examination. The larger lesion was 3.4 cm × 2.0 cm × 1.5 cm, and the smaller lesion was 2.0 cm × 1.3 cm × 0.9 cm. Cut sections demonstrated a smooth, yellow-brown to red-brown, and waxy appearance that was not well demarcated.

### Pathological and immunophenotypic findings

**Fig. 2:**
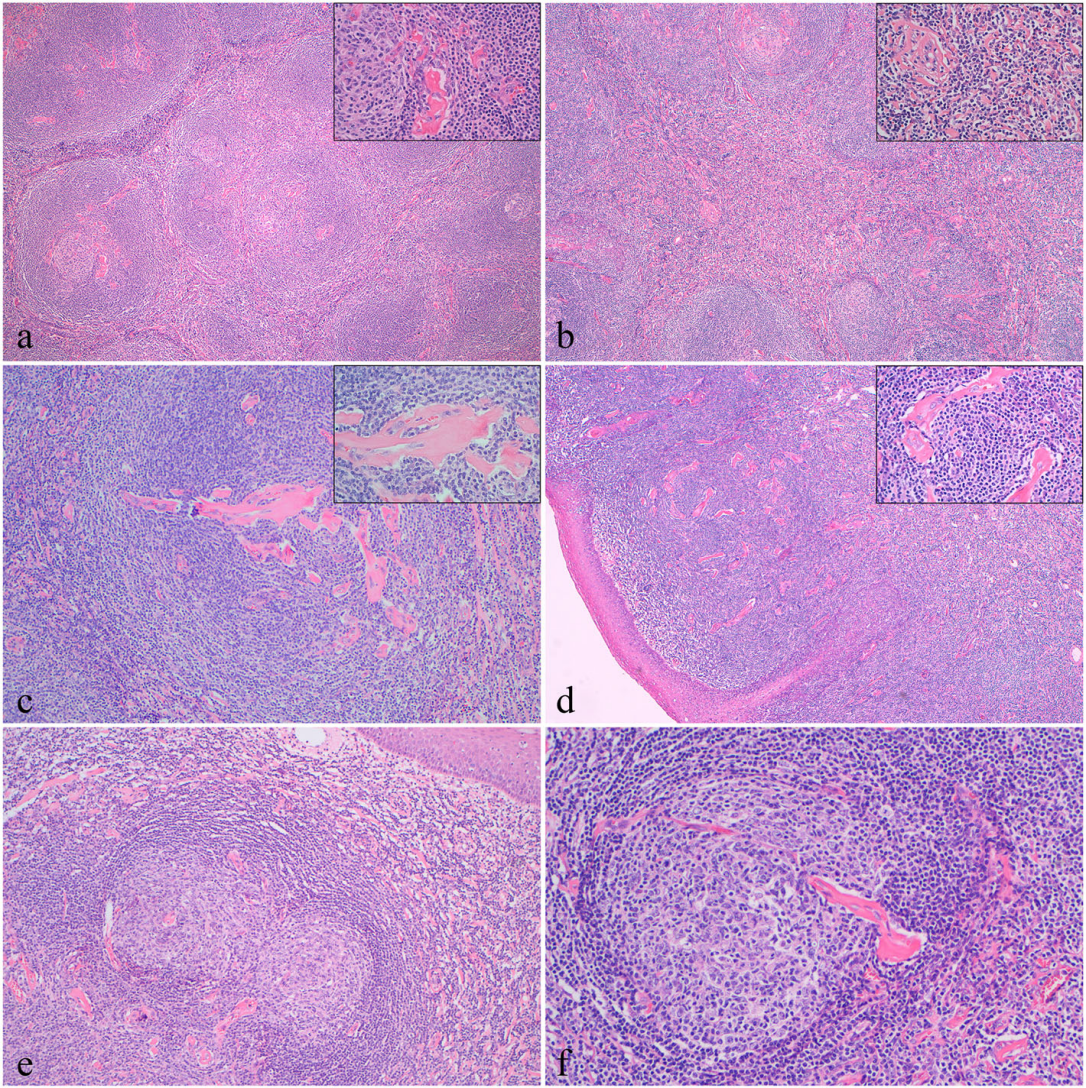
Histopathology of the tonsillar mass characteristic of HVCD (hematoxylin-eosin (H&E) staining). **a** (5× and 40× H&E): Photomicrographs showing lymphoid tissue with follicular hyperplasia, which was composed of lymphoid follicles of variable size and shape. These distinctive follicles had atrophic hyalinized germinal centers and a broad mantle zone. Increased numbers of thickened and hyalinized venules were observed in the center of lymphatic follicles and mantle zones. **b** (5× and 40× H&E): Many hyalinized venules in the interfollicular areas. **c** (10× and 40×, H&E) and **d** (5× and 40×, H&E): Germinal center atrophy even tended to disappear, and some areas were penetrated by small hyalinized vessels. **e** (5×, H&E): Lymphoid follicles with an expended mantle zone (onion-skin arrangement). **f** (5×, H&E): A characteristic lollipop appearance was observed (the onion-skin arrangement with a vessel penetrating the germinal center)

Sections of the tonsillar mass revealed the characteristic findings of HVCD. Microscopic examination of permanent sections showed polypoid masses unseparated from the surrounding normal tonsil, which were covered by well-differentiated squamous epithelium. No tonsil crypt structure was observed. Lymphoid follicular hyperplasia was the main pathologic finding, a portion of which appeared to be a fusion of nodular hyperplasia (composed of lymphoid follicles of variable size and shape) (Fig. 2a-c). These distinctive follicles with atrophic hyalinized germinal centers (depleted of centroblasts and centrocytes) and a broad mantle zone of small lymphocytes formed concentric rings (a so-called onion-skin arrangement). Both single follicles and confluent follicles with a single mantle zone were observed (Fig. 2a and e). Medium-sized vessels and a plethora of capillaries were present in the center of lymphatic follicles, mantle zones, and interfollicular areas (Fig. 2b-d). A characteristic lollipop appearance was also observed due to the onion-skin arrangement of the expanded mantle zone lymphocytes with a vessel penetrating the germinal center (Fig. 2f).

**Fig. 3:**
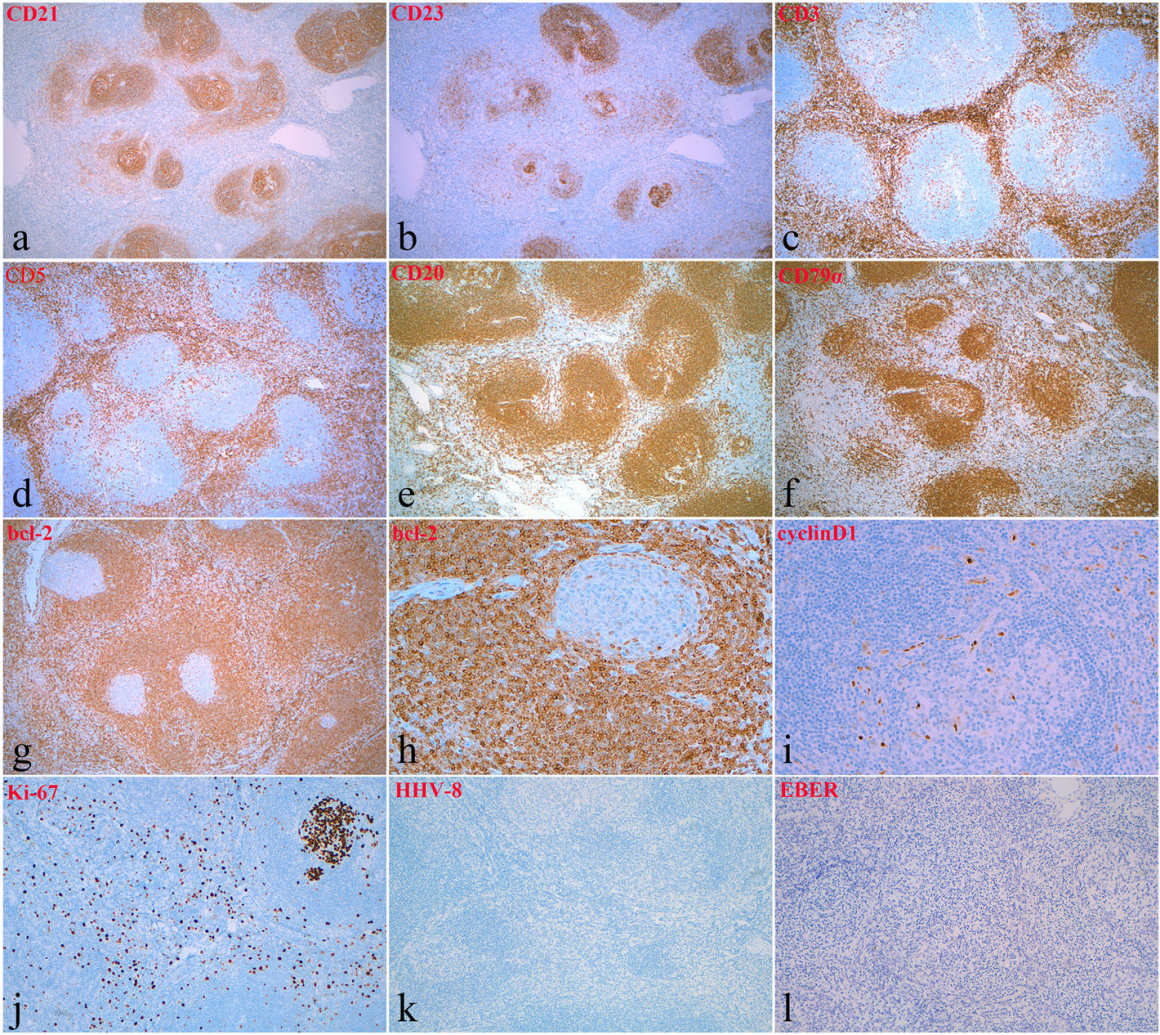
Immunohistochemical (IHC) features and in situ hybridization (ISH) results. **a** and **b** (5×, IHC): Follicular dendritic cells stained with CD21 and CD23 antibodies. **c** and **d** (5×, IHC): T-cells stained with CD3 and CD5 antibodies. **e** and **f** (5×, IHC): B-cells stained with CD20 and CD79α antibodies. **g** (5×, IHC) and **h** (20×, IHC): Bcl-2 staining indicated small and mature lymphocytes in the mantle zone and T-cells in the interfollicular areas. **i** (20×, IHC): The small lymphocytes in the expended mantle zone were negative for cyclin D1. **j** (10×, IHC): Ki-67 staining indicated proliferating cells, which were mainly observed in the germinal centers. **k** (10×, IHC): Immunostaining with HHV-8 was negative. **l** (10×, ISH): EBER-ISH to detect EBV was negative

To exclude the possibility of low-grade malignant lymphoma, a comprehensive immunostaining panel was performed. A meshwork of follicular dendritic cells in the germinal centers was highlighted by CD21 and CD23 staining (Fig. 3a and b). Cells constituting the expanded mantle zones expressed CD20 and CD79α (Fig. 3e and f). The B-cell population within both the follicles and interfollicular areas demonstrated polytypic expression of Ig light chains. The interfollicular areas were comprised predominantly of T-cells (immunoreactive for CD3, CD5, and bcl-2) admixed with scattered B-cells (immunoreactive for CD20, CD79a) (Fig. 3c-g). Bcl-2 staining also indicated small and mature lymphocytes in the mantle zone. The onion-skin arrangement was clearly visible via bcl-2 immunohistochemical staining (Fig. 3h). The small lymphocytes in the expanded mantle zone were negative for cyclin D1 (Fig. 3i). Ki-67 staining indicated proliferating cells, which were mainly observed in the germinal centers (Fig. 3j). Immunostaining with HHV-8 was negative (Fig. 3k). Epstein Barr virus (EBV) was not detected in the tonsillar lesion by in situ hybridization (ISH) for EBV-encoded small nuclear mRNA (EBER). EBER is an EBV-encoded small nuclear mRNA.

Based on these microscopic features and immunohistochemical findings, a diagnosis of HVCD was rendered. The patient was treated with local excision without any other therapy based on the diagnosis of HVCD. At the 7-month follow up, the patient had no recurrent symptoms or masses.

## Discussion and conclusions

Herein, we present a patient with extranodal HVCD located in the tonsil. Extranodal CD can be a challenging diagnosis, especially when it presents in an unusual location. This patient’s imaging exemplified some of the histopathological features that are typical of this diagnosis.

Generally, a definitive diagnosis for CD is established only by histologic examination of the surgical specimen [4]. Tissue that is obtained by fine needle aspiration or core needle biopsy is often nondiagnostic; therefore, an excisional biopsy is preferred. HVCD is characterized by distinctive follicles with regressed hyalinized germinal centers and a broad mantle zone of lymphocytes that form concentric rings (so-called onion-skin arrangement) [4]. Increased interfollicular vascularity with hyalinized vessels is another important feature. A characteristic lollipop appearance is demonstrated by the onion-skin appearance of the mantle zone lymphocytes with a vessel penetrating the germinal center [6, 8]. PCCD has fewer distinctive histologic features. However, it is characterized by the remaining lymph node architecture, variable hyperplastic germinal centers, interfollicular hypervascularity and marked PC sheet cytosis [4, 8]. The patient described in this report displayed all of the typical histopathologic characteristics of HVCD, such as atrophic hyalinized germinal centers with broad mantle zones of small lymphocytes that surround the germinal centers and interfollicular hypervascularity without PC sheets. Most importantly, our pathologic findings suggested a diagnosis of HVCD.

Differential diagnoses based on the gross and microscopic findings in this case mainly included low-grade malignant lymphomas, including follicular lymphoma and mantle cell lymphoma. Immunohistochemical assays revealing CD3 and CD5 positivity were consistent with the normal distribution of the T-cell population, and CD20 and CD79α positivity was consistent with the normal distribution of the B-cell population. No abnormality was found upon immunohistochemical staining for CD21 and CD23, which highlighted the follicular dendritic cells within the germinal centers. Ki-67 staining indicated approximately 90% positivity in the germinal center and approximately 3–5% positivity in the interfollicular areas. The expression pattern of ki-67 excluded the possibility of follicular lymphoma. Negative bcl-2 staining of regressed small germinal centers also excluded follicular lymphoma. Additionally, bcl-2 staining highlighted the expanded mantle zone. There were no cyclin D1-positive atypical lymphoid cells in the expanded mantle zone, which excluded mantle cell lymphoma. Therefore, a diagnosis of low-grade malignant lymphomas was excluded.

As previously mentioned, CD presents as either UCD or as MCD [2, 4]. Approximately 80–90% of UCD cases are classified as HVCD, while only 10–20% are classified as PCCD [4]. UCD is mostly asymptomatic and is diagnosed incidentally upon imaging. Symptoms may be present in some patients as a result of mass effects. While UCD can occur at any age, the median age of patients is 35 years, with an equal male/female ratio [7]. The majority of UCD cases originate in the mediastinum, lung, neck, pelvis, retroperitoneum and axilla. Most patients with MCD present with the PC type, and most have B symptoms (referring to nonspecific systemic symptoms of fever, night sweats, and weight loss). Peripheral lymphadenopathy is almost always present in MCD cases [3, 9]. Laboratory abnormalities of MCD patients include anemia, hypoalbuminemia, hypergammaglobulinemia, and elevated interleukin (IL)-6, erythrocyte sedimentation rate and C-reactive protein values [9, 10]. MCD typically presents in individuals between 50 and 65 years of age, although patients who are infected with HIV tend to be younger. Male patients are predominant (50–65%). A subset of patients experience skin rash, edema, body cavity effusion, and neurologic changes. Fewer than 10% of patients are asymptomatic [7–9]. The patient we describe herein is a young man who presented only a tonsil mass without any nonspecific findings in a physical examination. Laboratory tests also revealed no abnormal findings. Therefore, we believe that our patient had the HVCD type of UCD.

The etiopathogenesis of CD remains unclear. However, CD has been suggested to be associated with immunoregulatory defects in individuals infected with HHV-8 and HIV [5, 8]. HHV-8 has been shown to be involved in MCD, especially in PCCD, specifically in HIV-positive patients. These patients have a poor prognosis [8]. HHV-8 encodes viral IL-6, which can induce the secretion of endogenous human IL-6 and vascular endothelial growth factor and can also enhance angiogenesis [10]. Specifically, the role of HHV-8 and IL-6 in the pathogenesis of the PC variant of MCD has long been recognized. During symptomatic episodes, the serum IL-6 level is elevated in patients with the PCCD type [11]. Most studies suggest that neither EBV nor HHV-8 are involved in HVCD [12]. These results are consistent with our results. Immunostaining for HHV-8 and ISH for EBER were negative. Although HHV-8 infection would support a diagnosis of CD, a negative serologic or immunohistochemical assay for HHV-8 would not eliminate the diagnosis, especially for the diagnosis of HVCD. Therefore, this patient was diagnosed with HVCD mainly according to histologic findings.

Although extranodal CD will rarely be included in a list of differential diagnoses for lymphoproliferative disorders in the tonsil, it is important to recognize the occurrence of this disease because of its potential for transformation into follicular dendritic cell sarcoma. Mild to marked follicular dendritic cell dysplasia may be present within the follicles of HVCD masses [13]. In some cases of HVCD, it is possible to observe the proliferation of follicular dendritic cells outside of the follicles, forming clusters and sheets [6]. A cytogenetic study [14]. demonstrated a clonal karyotypic abnormality in an example of HVCD in the absence of histologic evidence of follicular dendritic cell sarcoma. A recent study has shown that some cases of HVCD could progress to follicular lymphoma [6]. Additionally, a study by Cokelaere K et al. proposed HVCD as a disease of follicular dendritic cells [15]. As previously mentioned, CD has also been associated with KS, non-Hodgkin’s lymphoma, Hodgkin’s lymphoma, HHV-8 and HIV. Therefore, accurate identification of extranodal CD is very important for the patient.

The treatment of patients with CD depends on its presentation. Surgical resection is curative for UCD [4]. Radiotherapy has been shown to improve the outcome of patients with UCD who have undergone an incomplete resection. Medical therapy such as steroid and combination chemotherapy is considered a primary treatment option for patients with MCD [16].

In summary, extranodal CD can be a challenging diagnosis to establish, especially when it presents in an unusual location. Herein, we described the microscopic features and immunohistochemical findings from an extremely rare case of CD that originated in an extranodal organ, the tonsil. Our patient presented with single lesions and did not show any systemic symptoms. This paper appears to be the first description of tonsillar HVCD. It is hoped that this report will prompt clinicians to consider CD when tonsillar masses are encountered.

## Data Availability

The datasets during and/or analysed during the current study available from the corresponding author on reasonable request.

## Abbreviations

CD: Castleman disease
EBER: EBV-encoded small RNA
EBV: Epstein Barr virus
HHV-8: Human herpesvirus-8
HIV: Human immunodeficiency virus
HVCD: Hyaline vascular Castleman disease
IHC: Immunohistochemical
ISH: In situ hybridization
KS: Kaposi’s sarcoma
MCD: Multicentric Castleman disease
PCCD: Plasma cell Castleman disease
PCs: Plasma cells
UCD: Unicentric Castleman disease
